# Clavicular augmentation with hyaluronic acid six-point technique: A novel non-surgical approach to skeletal definition

**DOI:** 10.1016/j.jpra.2025.12.005

**Published:** 2025-12-12

**Authors:** Kyu-Ho Yi, Isabella Rosellini, Jong Keun Song, Suyeon Lee, Carlos Bravo Rojas, Jin-Hyun Kim

**Affiliations:** a You and I Clinic, Seoul, Korea; b Avery Beauty Clinic And Avena Aesthetics, Jakarta, Indonesia; c Pixelab Plastic Surgery Clinic, Seoul, Korea; d Medical Research Inc., Wonju, Korea; e Private Practice, San José, Costa Rica

**Keywords:** Hyaluronic acid, Clavicular augmentation, Non-surgical approach, Body contouring, Aesthetic medicine, Case report

## Abstract

**Background:**

Clavicular augmentation using hyaluronic acid (HA) dermal fillers is an innovative aesthetic intervention for improving skeletal prominence through minimally invasive methods. Successful application requires a precise, structured technique targeting the subperiosteal plane.

**Case presentation:**

We describe a novel standardized technique—the six-point technique for non-surgical clavicular augmentation and present the outcome in a 35-year-old female patient.

**Technique/Methods:**

Under strict aseptic conditions, 2.0 mL of HA filler (Lorient No. 6) was injected per side into the subperiosteal plane using a fine-gauge needle. The Six-Point Technique involves structured, differential dosing to restore linear continuity (0.5 mL in concavities) and refine convex highlights (0.3 mL along curves).

**Results:**

The procedure resulted in immediate and sustained enhancement of the clavicular line and improved upper-torso contour. At the 3-month follow-up, the patient reported high satisfaction (9/10), with no severe adverse events observed. Documented complications remained predominantly minor and temporary, including localized inflammation or discomfort, without any severe adverse outcomes reported.

**Conclusions:**

The HA clavicular augmentation six-point technique is a safe, versatile, and reversible option for achieving predictable skeletal definition. This structured, minimally invasive treatment provides consistent, aesthetically pleasing outcomes, underscoring the potential for injectable solutions in skeletal contouring.

## Introduction

The clinical application of hyaluronic acid (HA) fillers has expanded rapidly in aesthetic medicine, supported by high-level evidence showing a generally favorable safety profile with mostly transient, mild to moderate adverse events (e.g., edema, erythema, ecchymosis) in appropriately selected patients.^1^ Recent systematic reviews and meta-analyses corroborate these findings, reinforcing HA’s role as a biocompatible, reversible option suitable for minimally invasive contouring applications beyond the face.^2^

In recent years, peer-reviewed reports have documented a shift toward body contouring indications, including reshaping with low G′ HA fillers and high G′ protocols for upper-body definition, demonstrating feasibility, patient satisfaction, and low complication rates in early follow-up. These publications align with broader clinical practice patterns favoring customizable, minimally invasive solutions that can be titrated by volume, plane, and rheology to achieve natural skeletal and soft-tissue definition without surgical downtime.^3^^,^^4^

The clavicular region presents unique anatomical considerations for minimally invasive aesthetic enhancement, given the superficial skeletal contour in proximity to the supraclavicular nerves and subclavian vessels, necessitating precise anatomical orientation, plane control, and conservative dosing to mitigate risk while optimizing aesthetic outcomes.^5^^,^^6^ This paper details a novel, standardized approach—the six-point technique—for HA-based clavicular augmentation, illustrating its application with a clinical case.

## Clinical case and technique

A 35-year-old woman presented requesting enhanced clavicular definition to achieve a slimmer, more sculpted upper-torso appearance following clinical evaluation and informed consent.

**Figure 1:**
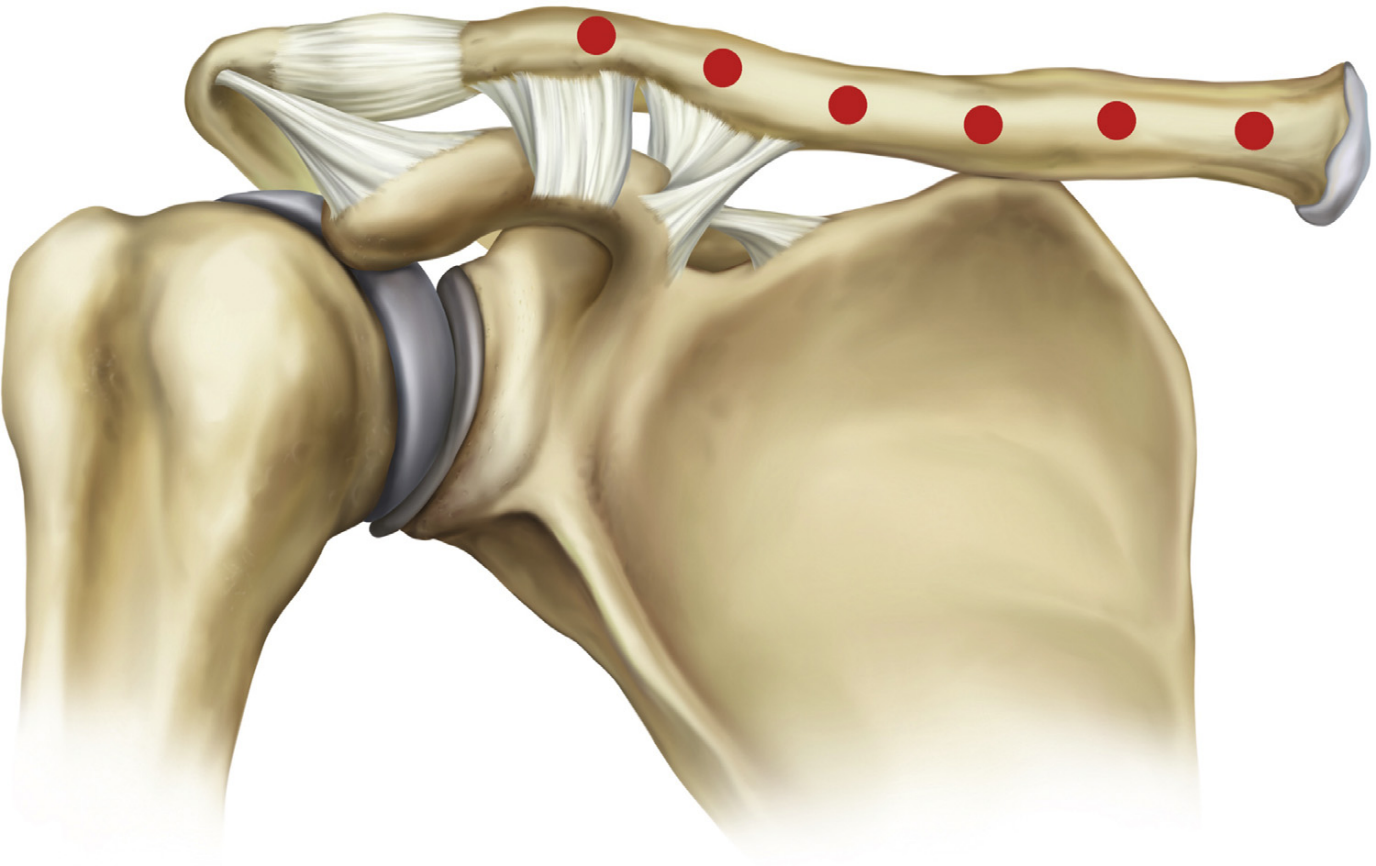
Schematic injection map and dosing allocation for minimally invasive hyaluronic acid augmentation of the clavicular line, showing planned deposition points and standardized volumes (approximately 0.5 mL in concavities; approximately 0.3 mL along forward-projecting curves).

Technique (Six-point technique): Under strict aseptic conditions, treatment was performed using a fine-gauge, short, sharp needle (25 G) inserted perpendicular to the skin and advanced slowly until the tip reached the periosteum. The syringe was then aspirated to ensure the needle was not in a blood vessel before a small bolus of filler was injected. This subperiosteal placement provided greater stability and was less prone to migration. The product used was Lorient No. 6 (Joonghun Pharmaceutical, South Korea), with a total of 2 mL administered per side. Approximately 0.5 mL was deposited into pre-marked concavities to restore linear continuity, while 0.3 mL was placed along forward-projecting curves to refine highlights without exaggerated convexity. Per-side totals were kept conservative at about 2.0 mL, with volumes adjusted to anatomy and aesthetic goals. Prior to injection, careful anatomical assessment and marking were performed to identify prominences and depressions while avoiding visible veins, and topical anesthesia was applied to minimize discomfort. Figure 1 provides a schematic injection map illustrating the standardized deposition points and volumes used for clavicular augmentation.

**Figure 2:**
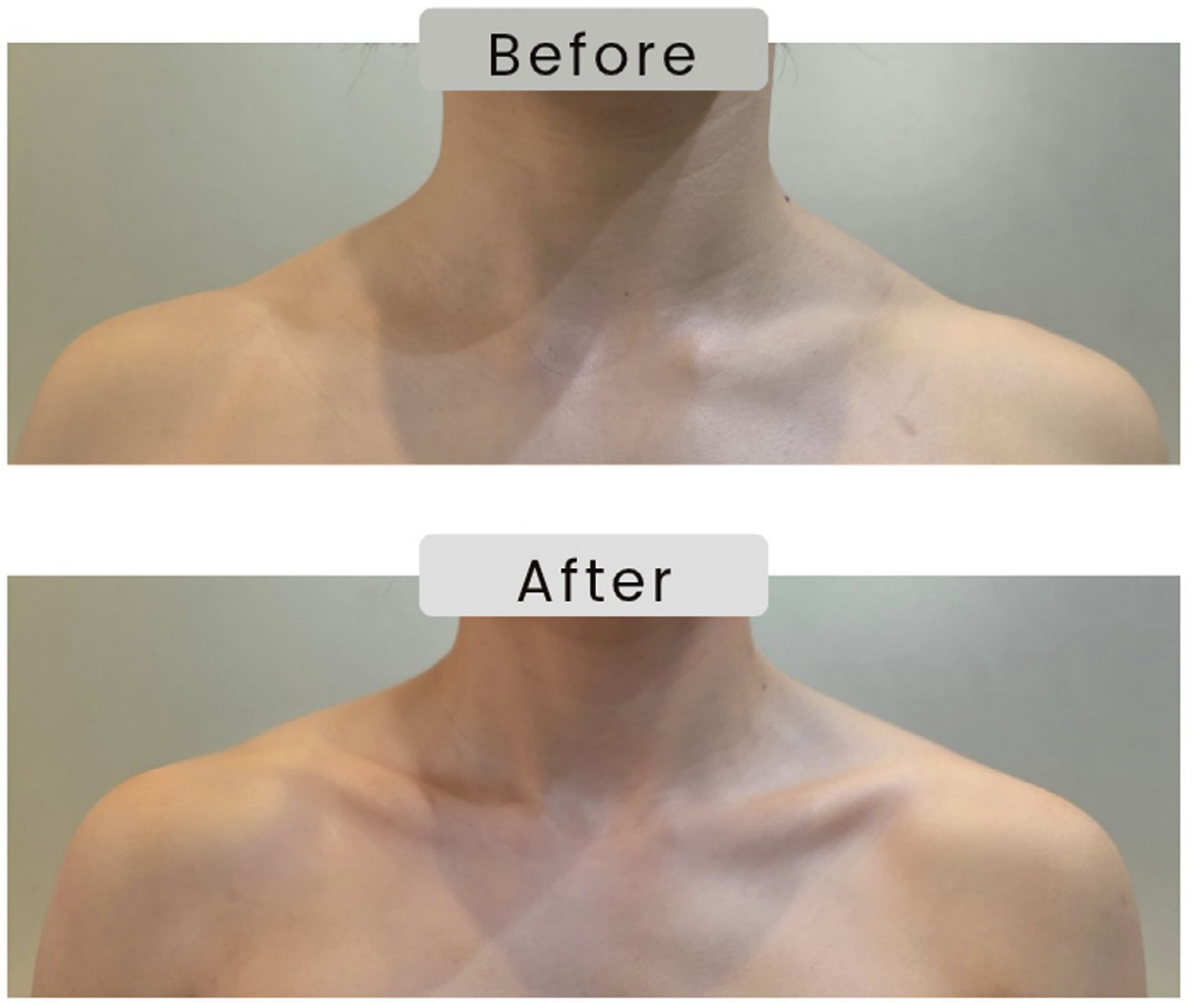
Standardized photographs of a 35-year-old woman obtained before (top) and immediately after (bottom) minimally invasive clavicular augmentation using hyaluronic acid filler (Lorient No. 6, Joonghun Pharmaceutical, South Korea). A total of 2 mL was administered per side, resulting in enhanced osseous definition and improved upper-torso contour.

Results and Follow-up: Immediate post-procedure evaluation demonstrated increased skeletal projection with more precise delineation of the clavicular line in the same-day assessment, and the patient reported high satisfaction, particularly regarding the perception of a slimmer upper-torso silhouette following treatment. Figure 2 presents comparative pre- and immediate post-treatment images that document greater clavicular prominence and refinement of the upper-body contour.

Post-injection care included the application of immediate pressure at the injection sites to minimize bruising and bleeding. The patient was instructed to avoid strenuous upper-body activity for several days and to refrain from touching or massaging the treated area unless specifically advised. A follow-up assessment was conducted at 3 months post-procedure. The results remained stable, with sustained aesthetic enhancement, and the patient reported a satisfaction score of nine out of 10.

## Discussion

The transition of HA fillers from facial indications to minimally invasive body contouring is supported by high-level evidence demonstrating a favorable safety profile and by recent syntheses that emphasize HA’s biocompatibility and reversibility, making clavicular refinement a logical, patient-centered application for proportionate skeletal definition with limited downtime.^1^^,^^2^ Within this evidence base, conservative enhancement of the clavicular line is clinically coherent as a means to improve upper-torso proportion without surgical morbidity when technique and dosing are standardized.^6^

Recent clinical advancements support the use of a subperiosteal approach for structural enhancement. Chang and Zhao demonstrated this via a linear technique along the clavicle, while Yi and Wan reported augmentation of the clavicle, acromion, and scapula with hyaluronic acid fillers to achieve a stable shoulder contour. Their findings emphasize that periosteal deposition improves skeletal definition and provides durable contouring outcomes without impairing muscular function.^6^^,^^7^ This reflects an emerging consensus that skeletal landmarks should guide injection strategies for long-lasting clavicular definition rather than soft tissue planes. This technique employs a point-lifting approach in which high–elastic modulus (high–G′) fillers (Lorient No. 6; Degree of Modification [MoD]: 2.239% ^1H, 1.947% ^13C; G′: 583 Pa; Joonghun Pharmaceutical) are deposited within the dense ligamentous and fibrous connective tissue complexes associated with the clavicular region. Lorient No. 6 is particularly well suited for this technique because its rheologic properties align with the biomechanical requirements of the clavicle-supporting ligamentous structures, providing adequate lifting capacity, structural reinforcement, and resistance to mechanical stress in this anatomically dynamic area.

The novelty of the six-point technique lies in its structured, multi-point, dual-dose framework. Unlike single-line or general augmentation techniques, this protocol systematically addresses distinct anatomical goals: the approximately 0.5 mL boluses are placed into mapped concavities to restore linear continuity, and the approximately 0.3 mL boluses are placed along forward projecting curves to specifically refine highlights. This targeted, volumetric strategy optimizes light and shadow balance, which is crucial for achieving natural skeletal definition.

Feasibility and early patient-reported benefits in body and upper-body contouring have been documented when product choice and incremental volumes are matched to structural aims, supporting protocolized, minimally invasive application to skeletal lines.^3^ The greatest advantage of clavicular augmentation with HA fillers is its safety and reversibility. HA is biocompatible and can be degraded by hyaluronidase in cases of overcorrection or complication, providing a significant safety margin compared to permanent surgical implants.^8^^,^^9^ Patients also benefit from a rapid procedure and short recovery time, often returning to daily activities within 24–48 h.^10^

The dosing framework applied here operationalizes those principles: approximately 0.5 mL is placed into mapped concavities to restore linear continuity and light–shadow balance, while approximately 0.3 mL is placed along forward-projecting curves to refine highlights, with typical per-side totals of about 1.0–2.0 mL and a preference for staged augmentation when further refinement is indicated.^4^^,^^6^

Notwithstanding its benefits, several constraints should be acknowledged. The clinical effect is temporary, typically persisting for approximately 6–12 months, contingent on product characteristics and patient-specific metabolic turnover. Consequently, periodic maintenance is often required and may increase cumulative costs. The clavicular region also entails distinct hazards due to its proximity to the subclavian vessels and pleura. Technical error can precipitate vascular compromise or unintended deep placement. Although uncommon, reported adverse effects include edema, ecchymosis, and localized tenderness.^6^^,^^11^

Best aesthetic gains are typically achieved with structurally supportive, high-lift HA gels capable of maintaining projection and contour stability in thin tissue regions.^3^^,^^6^

Placement immediately on bone remains the favored approach, with clinical experience indicating acceptable safety and durable line definition in carefully selected cases. Point-of-care ultrasound can serve as a useful adjunct to confirm the working plane in real time and to help avoid traversing vascular pathways during cannula passage.^5^^,^^6^

Safety in this area depends on strict attention to regional anatomy, given the clavicle’s proximity to the subclavian vessels and the superficial supraclavicular nerve branches.^5^ Risk mitigation includes using a blunt cannula in a bone-adjacent plane, limiting per-site volumes, and maintaining rigorous asepsis to reduce vascular, neural, and local complications such as edema or infection.^6^^,^^12^ If vascular compromise is suspected, rapid administration of hyaluronidase is recommended as the standard of care and patients should be counseled regarding the temporary nature of results and the likelihood of touch-up sessions.^13^

In summary, clavicular augmentation with HA filler represents a novel extension of skeletal contouring in aesthetic practice. By combining anatomical precision, advanced filler formulations, and minimally invasive technique, clinicians can achieve natural and predictable enhancement of the clavicular line.

## Conclusion

The growing demand for minimally invasive body contouring has positioned HA fillers as a versatile tool beyond traditional facial applications. Clavicular augmentation exemplified by the novel six-point technique, offering a non-surgical means of achieving skeletal definition that aligns with contemporary preferences for subtle, customizable, and reversible aesthetic enhancements. This case report highlights the feasibility and initial safety of the six-point technique, demonstrating high patient satisfaction and rapid recovery in this single patient. The results are encouraging; however, conclusions are limited to this initial case, and larger prospective studies are warranted to confirm long-term safety and efficacy of this novel indication.

## Ethical approval and consent to participate

Institutional review board (IRB) approval was deemed unnecessary as this work is a retrospective, single-patient case report detailing a novel technique. Written informed consent was obtained from the patient for the publication of clinical photographs and procedural details.

## Consent for publication

Written informed consent was obtained from the patient for publication of this case and accompanying images.

## Availability of data and materials

Not applicable.

## Funding

The authors received no financial support for the research, authorship, and/or publication of this article.

## Authors’ contributions

All authors contributed to the study conception and design. Material preparation, data collection, and analysis were performed by Kyu-Ho Yi, Isabella Rosellini, Jong Keun Song, Suyeon Lee, Carlos Bravo Rojas, and Jin-Hyun Kim. The first draft of the manuscript was written by Kyu-Ho Yi, and all authors commented on previous versions of the manuscript. All authors read and approved the final manuscript.

## Declaration of competing interest

The authors declare that they have no conflicts of interest to disclose.
